# Epicranial Arteriovenous Fistula: A Case Series and Review of the Literature

**DOI:** 10.7759/cureus.82776

**Published:** 2025-04-22

**Authors:** Miguel A Abdo Toro, Flavio Hernández González, Yair Ugalde Hernández, Pedro A González Zavala, Iván Téllez Medina, Carlos J Mávita Corral, Víctor R Chávez Herrera

**Affiliations:** 1 Department of Neurosurgery, Hospital de Especialidades, Centro Médico Nacional Siglo XXI, Instituto Mexicano del Seguro Social, Mexico City, MEX; 2 Department of Neurosurgery, Hospital General de León, León, MEX

**Keywords:** cirsoid aneurysm, endovascular therapy, epicranial arteriovenous fistula, scalp fistula, vascular neurosurgery

## Abstract

Epicranial arteriovenous fistulas (EAVFs) refer to vascular malformations located in the soft tissues of the scalp. They have a low incidence, and their cause can be traumatic, iatrogenic, or spontaneous. The arterial supply frequently originates from the superficial temporal artery and its branches. EAVFs present as a pulsating mass, with local pain and thrill, and sometimes occur as spontaneous bleeding. Six-vessel angiography is the gold standard for diagnosis, but AngioCT is also useful. The treatment options include surgery, embolization, or a combination of both. The decision for treatment should be made depending on the hemodynamics of the fistula. In this case series, we discuss four cases of EAVF: one male and three females, with a mean age of 37.8 years. Two of the fistulas were fed by the superficial temporal artery, one case by the occipital artery, and one case by both. Two cases required surgery and embolization, while two cases were treated with surgery alone. EAVFs are atypical events, and an appropriate understanding of their affluency, drainage, and hemodynamic features is required to decide the proper course of their management.

## Introduction

Epicranial arteriovenous fistulas (EAVFs), also referred to as scalp arteriovenous fistulas or cirsoid aneurysms, are a group of heterogeneous vascular lesions that occur infrequently in the general population, with only about 200 cases reported in 15 years [1,2]. They are anomalous connections between one or more arterial branches of the external carotid artery (typically branches of the superficial temporal artery) and a single venous channel without a capillary transition. The involved vessels are extracranial and located in the subcutaneous adipose tissue of the scalp [3]. The pressure difference between the arterial and venous systems causes venous dilation, leading to a pulsatile mass and other clinical symptoms, such as tinnitus or headache. Bleeding is uncommon, but it can occur and may even result in hemodynamic compromise [4].

The treatment of EAVFs can be challenging due to their complex and anomalous arterial and venous anatomy, high arterial irrigation, and aesthetic implications for the patient. These singularities influence the treatment modality. The treatment options include endovascular resection, sclerotherapy, and surgical resection. While endovascular approaches have gained ground in treating EAVFs, surgery is still mandatory in most cases to prevent recurrence [5].

There is a lack of awareness about this pathology in neurosurgical training because of its low frequency and approach by other surgical specialties. There are no established guidelines about this condition, and publications about it are predominantly limited to case reports. At our hospital, one of the main referral institutions in the country, EAVFs still represent an infrequent pathology, which can have an insidious evolution. Nevertheless, we present four EAVF cases successfully treated over the last 15 years at the "Hospital de Especialidades Centro Médico Nacional Siglo XXI (HECMNSXXI)” in Mexico City, Mexico. One of these cases manifested as active bleeding and had been treated previously in another center. We also provide recommendations about the treatment of this condition based on our experience.

## Case presentation

Case 1

**Figure 1:**
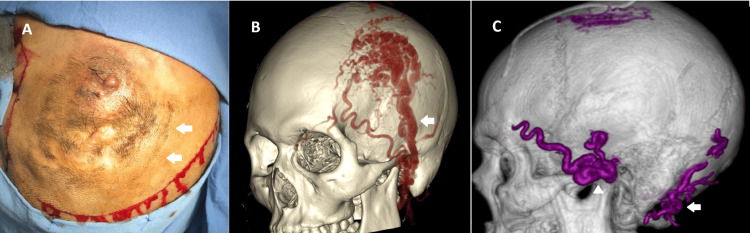
Left frontotemporoparietal EAVF A. Pulsatile and growing mass in the left frontoparietotemporal region. Surgical incision is visible. Scars from previous surgeries and bleeding are also visible (arrows). B. Preoperative AngioCT showing the afference of the STA (arrow) and occipital artery, a compact nest, and the tortuosity of the draining vessels. C. Postoperative AngioCT reconstruction shows no remnant of the fistula. The embolization agent is observed in the STA (arrowhead) and the occipital artery (arrow) EAVF: epicranial arteriovenous fistula; STA: superior temporal artery

A 31-year-old male with a smoking history presented with a bleeding left frontoparietal EAVF. He had suffered a contusion in the left frontoparietal region at the age of ten years; three months later, he had developed a pulsatile and painful mass associated with a thrill (Figure 1A). He had previously undergone unsuccessful surgical treatment twice at another center at the ages of 13 and 18 years. Over the past three months, he had experienced two minor episodes of bleeding and presented with 24 hours of active bleeding. On this occasion, he was referred to our center after losing a total of 2 liters of blood in 24 hours and having a transfusion. A six-vessel angiography revealed an EAVF supplied by the ipsilateral superficial temporal artery (STA) and the occipital artery, with a complex architecture, including a compact nest and tortuosity of the draining veins (Figure 1B).

The patient was scheduled for a combined and staged endovascular and surgical treatment. Initially, embolization was performed via transfemoral endovascular therapy using n-butyl-cyanoacrylate (Histoacryl), resulting in complete angiographic occlusion of the fistula after the procedure. Twenty-four hours later, a question-mark incision was made, followed by subcutaneous dissection of the lesion, coagulation, ligation, and resection of the afferent and efferent vessels. The postoperative recovery was successful, with the complete exclusion of the fistula, as demonstrated in the postoperative AngioCT (Figure 1C). The five-year follow-up showed no recurrence.

Case 2

**Figure 2:**
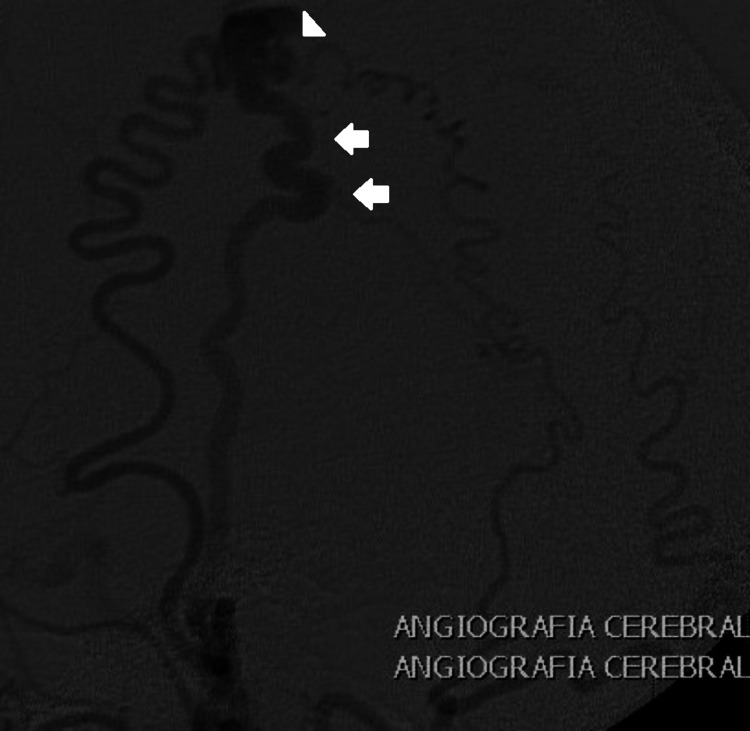
Frontal EAVF Lateral view of a six-vessel angiogram of a frontal EAVF displaying STA afference and a compact nest. Arrows: STA. Arrowhead: venous dilation EAVF: epicranial arteriovenous fistula; STA: superior temporal artery

A 46-year-old female with no history of trauma presented with a painful, pulsatile mass in the left frontal area. Physical exploration raised suspicion of a vascular lesion. The initial protocol included AngioCT in the referring hospital. An angiogram was performed at our center and revealed an EAVF with a compact nest and STA affluency (Figure 2). Surgical treatment was performed, consisting of a centered horseshoe incision, dissection and ligation of afferent and efferent vessels, and resection of the fistula. There was no recurrence at the five-year follow-up, and a total remission of the symptoms was observed.

Case 3

**Figure 3:**
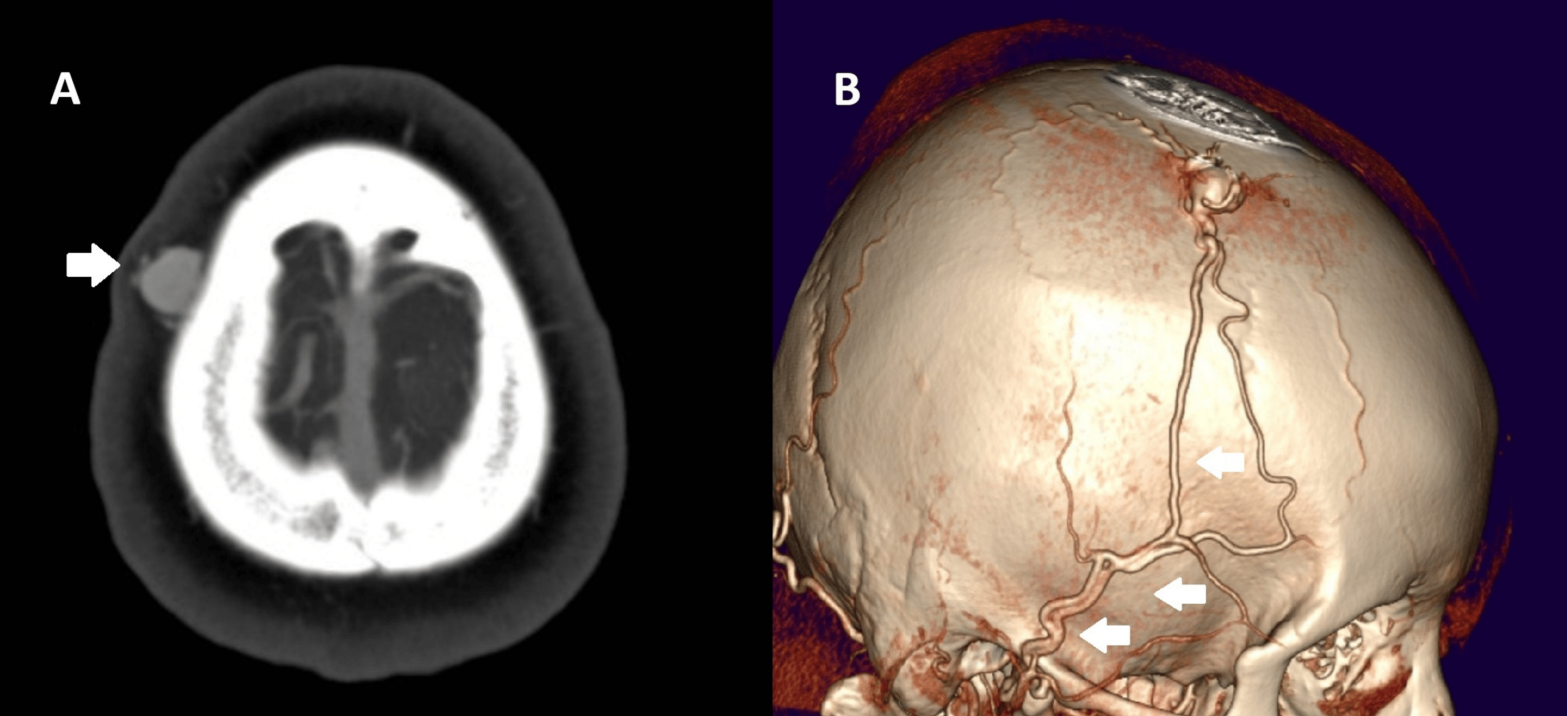
Right parietal EAVF AngioCT of a parietal EAVF displaying STA afference. A. Axial projection showing vessel dilation (arrow) and presence of mass in soft tissues. B. 3D reconstruction showing the vessel dilation and the arterial supply by STA (arrows) EAVF: epicranial arteriovenous fistula. STA: superior temporal artery

A 27-year-old female with no history of trauma presented with a pulsatile mass in the parietal region. An AngioCT revealed an STA-dependent EAVF with a venous dilation (Figure 3). She underwent surgical treatment, which led to favorable outcomes and complete excision of the lesion. The surgery consisted of a horseshoe incision, with ligation of involved vessels and resection of the fistula. At the five-year follow-up, there were no signs of recurrence.

Case 4

**Figure 4:**
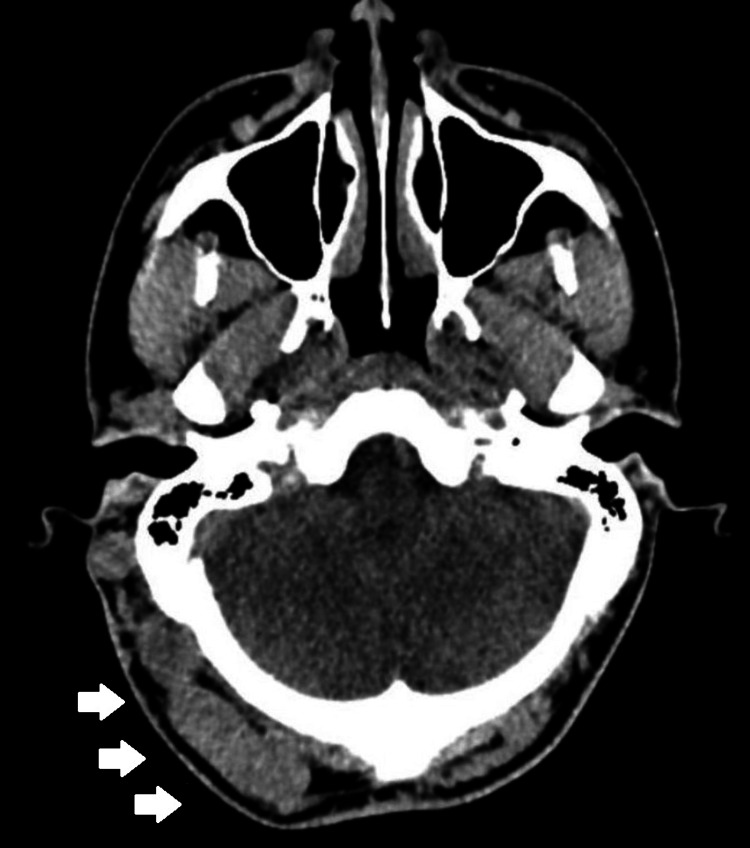
Right occipital EAVF Axial projection of a simple CT showing a soft tissue right parietooccipital mass, later diagnosed as an EAVF. Arrows show an isodense left parietooccipital lesion, which causes an increase in volume EAVF: epicranial arteriovenous fistula

A 47-year-old female, with a history of a contusion in the right parietooccipital region two years earlier, presented with a pulsatile mass in the right parietooccipital area (Figure 4), associated with local pain. An EAVF was identified in the angiogram, with an arterial supply from the occipital artery and muscular branches from the vertebral artery. The occipital artery was embolized with n-butyl-cyanoacrylate (Hystoacryl), followed by surgical treatment 24 hours later. Surgery consisted of a centered incision, dissection, and ligation of the patent afferent and draining vessels. No recurrence was observed at the five-year follow-up.

This series describes the cases of three females and one male, with an average age of 37.75 years. Two of the cases had a trauma history. Three of these cases presented with STA affluence, and half of the cases exhibited multiple afferent vessels. Two cases required embolization followed by surgery, while the other two were treated with surgery alone. None of the cases demonstrated recurrence at the five-year follow-up (Table 1).

**Table 1:** Summary of the cases One case presented as an active bleeding. Three cases received arterial supply from the STA, and two cases had multiple sources of supply. Surgery was the treatment option for all, although two also received embolization due to multiple afferent vessels. None of the cases showed recurrence at the five-year follow-up STA: superior temporal artery

Case	Gender	Age, years	Symptoms	Location	Arterial supply	Treatment	Five-year recurrence
1	Male	31	Bleeding, pain	Fronto-parieto-temporal	STA, occipital	Embolization + surgery	No
2	Female	46	Local pain, pulsatile mass	Frontal	STA	Surgery	No
3	Female	27	Pulsatile mass	Parietal	STA	Surgery	No
4	Female	47	Pulsatile mass, local pain	Parietooccipital	Occipital, the muscular branches of the vertebral artery	Embolization + surgery	No

## Discussion

EAVFs are very uncommon vascular malformations that involve the epicranial soft tissues [1,2]. They have been described using multiple names, including cirsoid aneurysm, scalp arteriovenous fistula, racemous aneurysm, and plexiform angioma, among others. They were first described by Bercht in 1833 [6]. EAVFs are abnormal direct connections between one or more arterial influences and typically a single venous draining channel. Due to their high flow, these connections can lead to tortuous, deformed, and disproportionately sized vessels. Unlike cerebral arteriovenous malformations, EAVFs communicate directly without the need to converge in a true capillary nidus, and they do not involve cerebral parenchyma. They also differ from dural arteriovenous fistulas because their arterial supply does not come from dural, cortical, or pial vessels.

The arterial supply mainly relies on branches of the external carotid artery, such as the STA, occipital artery, and posterior auricular artery [7,8]. Venous drainage occurs through superficial veins. The arteriovenous connection and the differences in pressure between the arterial and venous systems result in venous tortuosity and cutaneous deformity. In the cases presented, the arterial supply included the STA, and in half of the cases, it was multiple. It has a reported male predominance in males (2-2.5:1), with an average age of 25-29 years [7,8]. The literature review indicates an approximate prevalence of 200 cases reported over 15 years [9]. In the presented series, we found a female predominance and a slightly older age of presentation than previously reported, although one of the cases had been treated before and initially documented at a younger age (13 years).

Etiology can be divided into three main categories: iatrogenic, traumatic, and spontaneous. Traumatic EAVFs represent 32% of cases, with the specific etiology commonly including closed head trauma, contact sports, falls, contusions, motor vehicle accidents, gunshot wounds, and stabbings, among others. Reported cases most frequently develop two to five years after trauma [10]. Iatrogenic etiology accounts for 7.5% of total cases, typically occurring after procedures such as tumor or cyst resection, temporomandibular arthroscopy, capillary transplant, and craniotomies, and represents a rare complication. The majority of EAVFs are attributed to spontaneous etiology [8]. The data presented shows a similar distribution, with spontaneous fistulas being the most common, followed by traumatic fistulas.

The physiopathology of EAVFs is not fully elucidated, and several theories have been proposed. Among them, the theory of vascular laceration explains the formation of traumatic and iatrogenic EAVFs at the injured vascular crossing, facilitated by the secretion of angiogenic factors such as vascular endothelial growth factor (VEGF) and hypoxia-induced factor 1 (HIF-1), which promote endothelial proliferation, angiogenesis, and aberrant vascular connections [11]. Other theories include disruption of the vasa vasorum and persistence of congenital arteriovenous communications, which may better explain spontaneous EAVFs [12].

The most common clinical findings include headache, tinnitus, and cranial deformity, characterized by a classic triad of local growth, pain, and thrill, which disappears with manual compression of the STA [13]. New neurological deficits are uncommon [8,14,15]. Sofela et al. reported an incidence of pulsatile mass in 94% of patients with traumatic EAVFs, headache in 25%, and tinnitus in 20%, with symptoms developing between six days and 31 years after trauma, indicating a heterogeneous evolution [8]. Craniofacial trauma, pregnancy, and hormonal changes may exacerbate symptoms, thereby necessitating treatment.

The most common location of EAVFs is the frontal region, accounting for 24% of cases, followed by the temporal region at 17% and the occipital region at 15%. The predominant arterial supply comes from the STA in 79% of cases, with less frequent contributions from the posterior auricular artery and the occipital artery. In 45% of cases, the arterial supply is singular, while the remaining 55% have between two and seven arterial suppliers [8]. Among the cases presented, two involved the frontal region, while the other two pertained to the parietal and parietooccipital regions. Half of the patients exhibited a unique arterial supply, while the other half had multiple supplies.

AngioCT is crucial for describing the anatomy of the lesion and its relationship with the skull and epicranial tissues. The lesion appears as a flow void signal on MRI [16]. The six-vessel angiography with digital subtraction is the gold standard for diagnosing EAVF. It allows for evaluation of the size, arterial supply, and venous drainage of the EAVF [8,10]. It also helps to differentiate EAVF from other vascular malformations such as hemangiomas, cavernomas, and sinus pericrania [17]. Dural AVFs are part of the differential diagnosis and have different treatment approaches and considerations, which can lead to complications or recurrence [8]. Therefore, understanding of the arterial affluency in angiography is important for diagnosis and treatment. Other differential diagnoses include lymphatic malformations, lipomas, hematomas, cystic lesions, abscesses, lymphadenopathy, and soft tissue tumors [17,18].

Some classifications for EAVFs have been proposed [19]. Youkuichi proposed a classification based on the number of arterial suppliers of the fistula (A: single fistula with single arterial feeder, B: single fistula with multiple arterial fistula, and C: multiple fistulas with multiple plexiform feeding arteries with a single draining vein); with no clear correlation with the choice of treatment. Other classifications are more specific in describing the features of the fistula, but also with no clear applicability for treatment choice.

The treatment modalities include surgical resection and embolization. The choice of modality depends on the evaluation of the arterial supply, venous drainage, and anatomical relationships. Both modalities can be combined, and consultation for reconstructive surgery may be necessary in cases of deformity. Embolization is preferred when prior surgical procedures have been performed and in cases with multiple arterial sources [20]. For small lesions with distinct arterial supply, sclerotherapy is an appealing option [21]. Access for embolization has been described as transarterial, transvenous, and direct percutaneous; embolization materials include coils, Onyx, isobutyl-2-cyanoacrylate, n-butyl-cyanoacrylate, polyvinyl alcohol, and thrombin, among others [22]. Reported complications include transient pain, necrosis, residual mass [8], and embolization material migration [23,24].

Surgical resection provides complete removal of the lesion, resulting in a lower incidence of residual lesions or recurrence. It is advised to excise both supragaleal and subgaleal components of the EAVF [25]. The most common complications include wound infections and flap necrosis due to devascularization. In cases of tortuous access or complex anatomy, a two-phase treatment approach is a good option. It consists of an initial arterial ligation or embolization followed by surgical resection [21].

The presented cases are similar in features to the previously reported EAVFs in the literature. We consider that these cases show successful results in terms of recurrence at five years. In particular, this supports surgery as the basis for treatment, considering endovascular treatment as an adjuvant to facilitate surgery. Based on our experience, each case requires an individual analysis to determine the best treatment approach. Nevertheless, we endorse and prefer surgical treatment with or without prior embolization, as it assists in achieving a more complete resection of the lesion with a lower risk of recurrence. Embolization should be considered when multiple feeders exist to minimize surgical bleeding and facilitate the dissection of the fistula. Complex or deforming EAVFs should be assessed for reconstructive surgery to determine the necessity of tissue expanders or skin flaps to achieve a satisfactory cosmetic result.

## Conclusions

EAVFs are rare vascular malformations that are most frequently spontaneous, although they can be associated with trauma or medical procedures. Six-vessel angiography is the gold standard for diagnosis and treatment planning, but AngioCT and CT can also be useful. Treatment options include surgical intervention, embolization, or a combination of both. The choice of treatment should consider anatomic and hemodynamic features. In particular, we recommend surgical treatment as the main option to be considered, with previous endovascular embolization in the case of multiple feeders.
